# Expressions of MUC1 and vascular endothelial growth factor mRNA in blood are biomarkers for predicting efficacy of gefitinib treatment in non-small cell lung cancer

**DOI:** 10.1186/1471-2407-14-848

**Published:** 2014-11-19

**Authors:** Jian Li, Yi-Ming Hu, Yong-Jie Du, Li-Rong Zhu, Hai Qian, Yan Wu, Wei-Lin Shi

**Affiliations:** Department of Pulmonary Medicine, Affiliated Hospital of Jiangsu University, 438 North Jiefang Street, Zhenjiang, 212001 China; Institute of Medical Science, Jiangsu University, Zhenjiang, China

**Keywords:** Non-small cell lung cancer, Gefitinib, MUC1, Vascular endothelial growth factor, Treatment response, Survival

## Abstract

**Background:**

Gefitinib, an EGFR-tyrosine kinase inhibitor, significantly improve prognosis in patients with advanced non-small cell lung cancer (NSCLC). The aim of this study was to evaluate the usefulness of MUC1 and vascular endothelial growth factor (VEGF) mRNA expression in peripheral blood as means of predicting benefit from gefitinib therapy in NSCLC patients.

**Methods:**

MUC1 and VEGF mRNA expressions were detected in peripheral blood of 66 patients with advanced NSCLC before (B0) and 4 weeks after treatment (B4w) with gefitinib, using real-time quantitative-PCR assay. Correlations between blood MUC1 and VEGF mRNA expression at B0 and B4w and the response to gefitinib treatment and survival were analyzed.

**Results:**

Blood levels of MUC1 and VEGF mRNA at B0 and at B4w were significantly higher in patients with progressive disease than in those with partial response and stable disease. Furthermore, blood MUC1 and VEGF mRNA positivity at two time points were strongly associated with shorter progression-free survival (PFS) and overall survival (OS) (P = 0.005 and P = 0.008 at B0, and P < 0.001 and P = 0.001 at B4w, respectively, for MUC1; P = 0.004 and P = 0.009 at B0, and P = 0.001 and P < 0.001 at B4w, respectively, for VEGF). Multivariate analyses demonstrated that blood MUC1 and VEGF mRNA positivity at B0 and B4w were independent factors for predicting worse PFS and OS.

**Conclusions:**

MUC1 and VEGF mRNA positivity in blood seem to be indicators of unfavorable response and poor PFS and OS in patients with advanced NSCLC treated with gefitinib and may be promising noninvasive and repeatable markers for predicting efficacy of gefitinib treatment.

## Background

Lung cancer is the leading cause of cancer death worldwide and it is responsible for more than 1 million deaths annually [1]. Almost 85% lung cancer can be classified as non-small cell lung cancer (NSCLC), with 65% to 75% of case presenting as locally advanced (stage III) or metastatic disease (stage IV) [2, 3]. Chemotherapy is associated with modest survival benefit and improved quality of life [4, 5]; however, its efficacy has clearly reached a plateau, and thus further improvements will require integration of novel therapies. Among the target agents, epidermal growth factor receptor (EGFR) inhibitors gefitinib and erlotinib are now established as an option for first-, second- or third-line treatment, or as maintenance treatment [6–11].

Considerable research has been undertaken to identify molecular markers that predict sensitivity to EGFR-tyrosine kinas inhibitors (TKIs). On the basis of the data from clinical trials comparing EGFR-TKIs with placebo or chemotherapy, EGFR-activating mutation status appears to be the most valid marker for the selection of patients who would derive the most benefit from EGFR-TKI treatment [7–9, 12–14]. Nevertheless, the clinical efficacies of EGFR-TKIs differ among such patients, and almost all individuals eventually develop resistance to these drugs. Moreover, clinical studies have also shown that even in patients with wild-type EGFR, EGFR-TKIs are either superior to placebo or not inferior to docetaxel chemotherapy as a second- or third-line therapy [9, 10]. To date, no effective biomarker is currently available for patients with wild-type EGFR tumor [15]. In addition, it is sometimes difficult to obtain sufficient tumor samples from patients with inoperable NSCLC for mutation analysis. Hence, practical clinical studies using blood markers that can predict treatment efficacy of NSCLC to EGFR-TKIs are urgently required.

Some studies have reported that serum levels of MUC1 (mucin 1, also called KL-6) and vascular endothelial growth factor (VEGF) are associated with tumor response, progression-free survival (PFS) and overall survival (OS) in NSCLC patients treated with EGFR-TKIs [16, 17]. Blood samples can be obtained safely, with the option of repeat sampling from all NSCLC patients regardless of patient characteristics. In this report, we prospectively studied the expression levels of MUC1 and VEGF mRNA in peripheral blood of patients with advanced NSCLC who underwent treatment with gefitinib. The aim of this study was to identify whether there are correlation between MUC1 and VEGF mRNA levels in blood of these patients and both response to gefitinib and survival benefit from gefitinib.

## Methods

### Patients

In this prospective study, patients aged ≥20 years with histologically confirmed stage IIIB or IV NSCLC in whom one or two prior chemotherapy regimen had failed or who were unsuitable or unwilling to undergo such chemotherapy were eligible for study inclusion. Patients were required to have tumor tissue accessible for tissue sampling by bronchoscopy, or lymph node biopsy (metastatic sites), or surgery; clinically measurable disease; performance status (PS, according to the criteria of Eastern Cooperative Oncology Group) of 0 to 3; adequate bone borrow, renal and hepatic function and an interval of ≥4 weeks since previous surgery or radiotherapy. All patients received gefitinib 250 mg orally once a day until disease progression, patient refusal, or development of intolerable toxicity, or death. This study was performed in accordance with the Declaration of Helsinki and has been approved by the ethic committee of Affiliated Hospital of Jiangsu University in China. Written informed consent was obtained from all participants.

### Study design

All patients had a pretreatment tumor assessment by computerized tomography (CT) scan, which was repeated to assess tumor response after a maximum of 8 weeks from the beginning of the treatment, then every 2 months until 9th month, and every 4 months thereafter. Tumor response was evaluated using the criteria of RECIST [18], classified as a complete response (CR), a partial response (PR), stable disease (SD), or progressive disease (PD). CR and PR were defined as the objective response. Disease control was judged when patients achieved the best response of CR, PR, or SD, which was confirmed and sustained for 6 weeks.

### Specimen collection

For all NSCLC patients, blood specimens were collected within one week prior to (B0) and 4 weeks after the start of gefitinib administration (B4w). Meanwhile, blood samples of 55 patients with benign lung disease (BLD) were used as controls. BLD included chronic obstructive pulmonary disease (18), asthma (14), pneumonia (12), interstitial lung disease (6), tuberculous pleurisy (5).

Approximately 6 mL peripheral blood from all of the subjects was collected into EDTA-containing tubes, stored at 4°C, and processed within two hours. The first 4 mL of peripheral blood collected were discarded to avoid contamination with skin epithelial cells. Peripheral blood mononuclear cells (PBMCs) were firstly isolated by density centrifugation (1500 rpm for 15 min) with lymphocyte separation medium and washed with PBS (1200 rpm for 10 min), cell pellet were suspended in 1 mL of Isogen (Nippon Gene, Toyama, Japan) and were stored at -80°C until use.

### RNA isolated and real-time quantitative-PCR

Total RNA was extracted by the guanidium-isothiocyanatephenol-chloroform-based method. The purity and quality of the RNA were measured by UV-visible spectrophotometer (Bio-Tek); 2% agarose gel electrophoresis and ethidium bromide staining were used to assess the integrity of the obtained RNA. First-strand cDNA was produced from total RNA by using an RNA PCR kit version 3.0 (TakaRa Bio Inc., Tokyo, Japan), according to manufacturer’s instruction.

The real-time quantitative (RTQ)-PCR of MUC1 and VEGF gene and β-actin as internal control was carried out on an ABI 7500 thermal cycler Real-time PCR system (Applied Biosystems, Foster Cyty, CA, USA), using the SYBR-Green I chemistry. Amplification primers of the three genes were synthesized by BioAsia Corporation (Shanghai, China) as follows: primer sequences for MUC1 were 5’AATGAATGGCTCAAAACTTGG3’ and 5’CACTAGGTTCTCACTCGCTCAG3’ and for VEGF, 5’GAGTACATCTTCAAGCCATCCTG3’ and 5’TGCTCTATCTTTCTTTGGTCTGC3’, and for β-actin, 5’TGACGTGGACATCCGCAAAG3’ and 5’CTGGAAGGTGGACAGCGAGG3’. The cycling conditions have been described in detail in previous report [19]. Detection of PCR products was accomplished by measuring the emitting fluorescence (Rn) at the end of each reaction step. Threshold cycle (Ct) correspond with the cycle number required to detect a fluorescence signal above the baseline.

Relative quantification was calculated with the Ct (2^—△△Ct^) method [20]. Each experiment was performed in triplicate. The average value of the replicates was used as quantitative value for each sample.

### Detection of EGFR mutation

One tumor biopsy or surgery sample from each patient was snap frozen immediately in liquid nitrogen. DNA was extracted from tissue samples containing more than 70% tumor cells using the QIAamp DNA Mini kit (Qiagen, Hilden, Germany). EGFR mutations in exon 18 to 21 were detected by PCR based direct sequencing reported previously [21]. The primers used and amplification condition have been described in detail [21]. PCR products were 2% gel-purified with a QIA gen gel extraction kit (Qiagen). DNA templates were processed for the DNA sequencing reaction using the ABI-PRISM Big Dye Terminator version 3.1 (Applied Biosystems, Foster Cyty, CA) with both forward and reverse sequence-specific primer according to the manufacturer’s guidelines. Sequence data were generated with the ABI PRISM 3100 DNA Analyzer (Applied Biosystems). Sequences were analyzed by Sequencer 3.1.1 software (Applied Biosystems) to compare variations.

### Statistical analysis

Blood MUC1 and VEGF mRAN levels are presented as median (range) because they were not normally distributes. Differences in the levels of both markers before treatment compared with 4 weeks after treatment and differences in patients with a PR or SD compared with those with PD were analyzed by Mann–Whitney test. The relation between MUC1 and VEGF mRNA levels was assessed using the spearman correlation coefficient. Associations between MUC1 or VEGF mRNA positivity and clinicopathologic factors including response to treatment were examined by Fisher’s exact tests. PFS was defined as the interval between the start of gefitinib therapy and the first manifestation of PD or death from any cause. OS was defined as the interval between the start of gefitinib therapy and death from any cause. The survival curves for PFS and OS were estimated using the Kaplan-Meier method, and differences between the two groups were compared with the log-rank tests. Multivariate Cox proportional hazard model was applied to examine whether the positive expressions of MUC1 or VEGF mRNA in blood were associated with PFS of OS even after adjustment for other prognostic factors. All tests were two sided, and P value <0.05 was considered statistically significant.

## Results

### Patient characteristics and treatment response

A total of 66 patients were enrolled this study. The patient characteristics are shown in Table 1. Twenty nine (43.9%) patients were female and 22 (33.3%) were never-smokers, with the median age of all patients being 67 years (range, 42–79 years). Thirty nine (59.1%) had adenocarcinoma, 52(78.8%) had PS 0–1, and 20 patients (30.3%) received gefitinib as first-line therapy. A total of 60 tumor samples were suitable for EGFR mutation analysis. EGFR mutations were identified in 22 (33.3%) of the 60 patients, 14 patients had deletions mutations in exon 19 and 8 patients had the point mutations in exon 21 (L858R). The results for response to gefitinib showed that 25 patients (37.9%) achieved a PR, and 20 (30.3%) had SD. The other 21 patients (31.8%) had PD. The response rate (PR) was 37.9%, the disease control rate (PR + SD) was 68.2%. Regarding association between treatment response and clinicopathologic factor, female gender (P = 0.007), adenocarcinoma histology (P = 0.004) and an EGFR mutation status (P = 0.005) were significantly associated with disease control rate achieved by gefitinib treatment (Table 1). In addition, adenocarcinoma (P = 0.022) and EGFR mutation (P = 0.018) were significantly associated with the responsiveness to gefitinib, but no association was found between other clinicopathologic factors and the response to gefitinib therapy (Table 1).

**Table 1:** **Associations between patient clinicopathologic factors and response to gefitinib**

Variable	Total (n = 66) (%)	Response to gefitinib treatment				
		PR (N = 25)	SD (n = 20)	PD (n = 21)	P value (PR vs SD + DP)	P value (PR + SD vs PD)
Sex						
Male	37(56.1)	11	9	17	0.136	0.007
Female	29(43.9)	14	11	4		
Age, yr						
<67	34(51.5)	11	11	12	0.447	0.603
≥67	32(48.5)	14	9	9		
Smoking history						
Never	22(33.3)	11	7	4	0.187	0.112
Former	18(27.3)	6	5	7		
Current	26(39.4)	8	8	10		
Histology						
ADC	39(59.1)	20	12	7	0.022	0.004
SCC	22(33.3)	4	6	12		
ASC	5(7.6)	1	2	2		
Performance status						
0-1	52(78.8)	19	16	17	0.760	0.421
2-3	14(21.2)	6	4	6		
Disease stage						
IIIB	10(15.2)	5	2	3	0.729	0.945
IV	56(84.8)	22	16	18		
Prior chemotherapy						
Yes	46(69.7)	18	16	12	0.790	0.157
No	20(30.3)	7	4	9		
EGFR status						
Wild-type	38(57.6)	9	11	18	0.018	0.005
Mutation	22(33.3)	12	8	2		
Unknown	6(9.1)	4	1	1		
MUC1 mRNA at B0						
Median	5.95	4.87	5.18	8.76	0.116	0.005
Range	1.16-17.56	1.16-8.75	2.45-10.73	2.24-17.56		
VEGF mRNA at B0						
Median	4.88	4.21	4.72	7.65	0.122	0.004
Range	1.07-15.32	1.07-9.86	1.62-10.24	2.31-15.32		

PR, partial response; SD, stable disease; PD, progressive disease; ADC, adenocarcinoma; SCC, squamous cell carcinoma; ASC, adenosquamous cell carcinoma.

### Analyses of MUC1 and VEGF mRNA levels in blood specimens of NSCLC patients

The blood levels of MUC1 and VEGF mRNA in NSCLC patients before (B0) and 4 weeks after gefitinib treatment (B4w) were significantly higher than in BLD patients (Table 2). Moreover, the blood levels of MUC1 and VEGF mRNA markedly decreased after treatment (Table 2). Meanwhile, a correlation was found between MUC1 and VEGF mRNA levels in blood sample (spearman correlation analysis: r_s_ = 0.538, P = 0.003).

**Figure 1:**
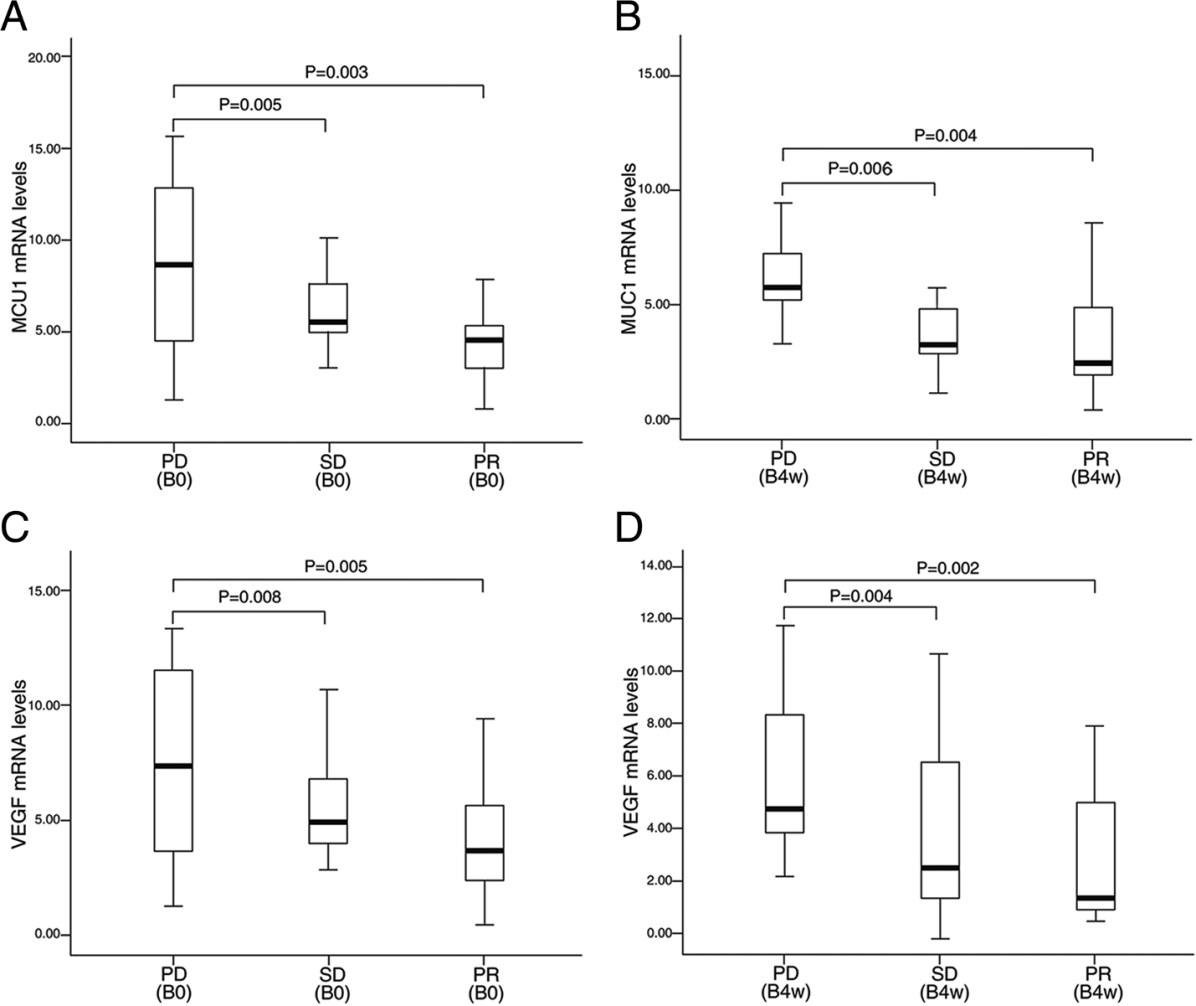
**MUC1 and VEGF mRNA levels in blood of NSCLC patients. (A and B)** Box-whisker plots of blood MUC1 mRNA levels in NSCLC patients with progressive disease (PD), stable disease (SD) and partial response (PR) before (B0) and 4 weeks after the start of gefitinib treatment (B4w). **(C and D)** Box-whisker plots of blood VEGF mRNA levels in NSCLC patients with PD, SD and PR at B0 and B4w.

Figure 1 shows associations between the blood levels of MUC1 and VEGF mRNA and response to treatment. At B0 and B4w time points, MUC1 and VEGF mRNA levels in patients with PR or SD were significantly lower than those in patients with PD (PR vs PD, P = 0.003; SD vs PD, P = 0.005, respectively at B0; PR vs PD, P = 0.004; SD vs PD, P = 0.006, respectively at B4w) (Figure 1A and B). Similarly, VEGF mRNA levels at two time points were significantly lower among patients with PR or SD than among those with PD (PR vs PD, P = 0.005; SD vs PD, P = 0.008, respectively at B0; PR vs PD, P = 0.002; SD vs PD, P = 0.004, respectively at B4w) (Figure 1C and D). No difference was observed in the levels of MUC1 and VEGF mRNA between patients with PR and those with SD.

**Figure 2:**
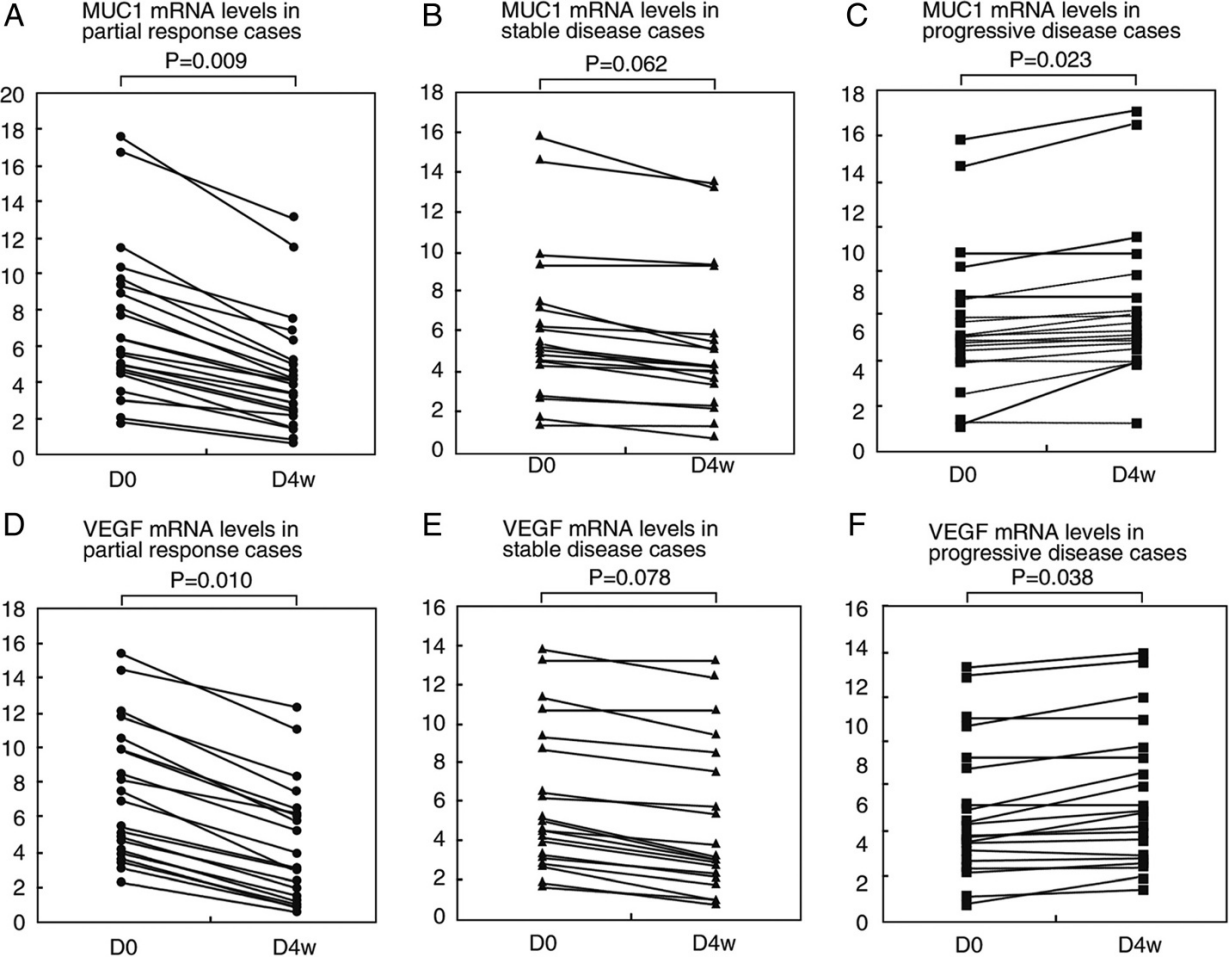
**Changes of MUC1 and VEGF mRNA levels in blood of NSCLC patients. (A to C)** Changes of MUC1 mRNA levels between before (B0) and 4 weeks after gefitinib treatment (B4w) in blood of NSCLC patients with partial response (PR), stable disease (SD) and progressive disease (PD). **(D to F)** Changes in blood VEGF mRNA levels between B0 and B4w time points in NSCLC patients with PR, SD and PD.

Figure 2 shows the changes in blood levels of MUC1 and VEGF mRNA in patients with PR, or SD, or PD, before and 4 weeks after gefitinib treatment. In the patients with PR, MUC1 and VEGF mRNA levels at B4w were significantly lower as compared with those at B0 time point (P = 0.009 and P = 0.010, respectively) (Figure 2A and D). In patients with SD, MUC1 and EVGF mRNA levels at D4w were marginally lower than those at B0 (P = 0.062 and P = 0.078, respectively) (Figure 2B and E). In the patients with PD, however, the two marker mRNA levels at B4w were significantly higher than those at B0 (P = 0.023 and P = 0.038, respectively) (Figure 2C and F)

**Table 2:** **Blood levels of MUC1 and VEGF mRNA in NSCLC patients and BLD patients at two sampling time points**

Patient	N	MUC1 mRNA					VEGF mRNA				
		B0		B4w		P value	B0		B4w		P value
		Median	Range	Median	Range		Median	Range	Median	Range	
NSCLC	66	5.95	1.16-17.56	4.46	0.64-12.45	0.027	4.88	1.07-15.32	3.82	0.88-13.84	0.035
BLD	55	2.06	0.32-4.17				1.75	0.41-3.28			
P value		<0.001		0.002*			<0.001		0.003*		

*NSCLC patients at B4w compared with BLD patients.

### Association between MUC1 and VEGF mRNA positivity and clinicopathologic factors

The maximum values of MUC1 and VEGF mRNA in BLD patients were 4.17 and 3.28 respectively (Table 2). Thus, the cutoff values of 4.2 and 3.3 were used as positive threshold for MUC1 and VEGF mRNA respectively. The blood samples were regarded as MUC1 or VEGF mRNA positivity if MUC1 and VEGF mRNA level above the two cutoff values respectively. Using the two cutoff values, 75.8% (50/66) and 45.5% (30/66) of B0 and B4w blood samples were considered MUC1 mRNA positivity; 71.2% (47/66) and 43.9% (29/66) of B0 and B4w blood samples were considered VEGF mRNA positivity, respectively. The positive rates of the two markers were significantly lower at B4w as compared with at B0 (P = 0.001 and P = 0.003, respectively).

In this study, we analyzed associations between MUC1 and VEGF mRNA positivity at B0 and B4w and clinicopathologic factors. As shown in Table 3, histology (P = 0.045 and P = 0.024, respectively) and the response to gefitinib treatment (P = 0.013 and P = 0.002, respectively) were significantly associated with MUC1 mRNA positivity at B0 and B4w. Similarly, the associations were found between the VEGF mRNA positivity at two sampling time points and histology (P = 0.053 and P = 0.013, respectively) and response to gefitinib treatment (P = 0.025 and P = 0.018, respectively). In addition, EGFR mutation status seems to be associated with MUC1 or VEGF mRNA positivity, even though these differences were borderline statistically significant (Table 3). No association was found between the MUC1 or VEGF mRNA positivity and other clinicopathologic factors.

**Table 3:** **Associations between MUC1 or VEGF mRNA positivity and clinicopathologic factors**

	MUC1 mRNA				VEGF mRNA			
	B0		B4w		B0		B4w	
	Positivity (n = 50)	Negativity (n = 16)	Positivity (n = 30	Negativity (n = 36)	Positivity (n = 47)	Negativity (n = 19)	Positivity (n = 29)	Negativity (n = 37)
Sex								
Male	27	10	16	21	25	12	17	20
Female	23	6	14	15	22	7	12	17
P value	0.579		0.804		0.586		0.805	
Smoking history								
Never	15	7	7	15	13	9	7	15
Non-never	35	9	23	21	34	10	22	22
P value	0.225		0.121		0.156		0.168	
Histology								
ADC	26	13	13	26	24	15	12	27
Non-ADC	24	3	17	10	23	4	17	10
P value	0.045		0.024		0.053		0.013	
Performance status								
0-1	40	12	22	30	36	16	22	30
2-3	10	4	8	6	11	3	7	7
P value	0.730		0.375		0.741		0.763	
Disease stage								
IIIB	7	3	6	4	5	5	6	4
IV	43	13	24	32	42	14	23	33
P value	0.695		0.814		0.248		0.437	
EGFR status								
Wild-type	32	6	21	17	31	7	21	17
Mutation	13	9	6	16	12	10	7	15
Unknown	5	1	3	3	4	2	1	5
P value	0.083		0.102		0.058		0.095	
Tumor response								
PR	14	11	7	18	13	12	8	17
SD	17	3	7	13	16	4	6	14
PD	19	2	16	5	18	3	15	6
P value	0.013		0.002		0.025		0.018	

ADC, adenocarcinoma; PR, partial response; SD, stable disease; PD, progression disease.

### Correlation between MUC1 and VEGF mRNA positivity and PFS and OS

**Figure 3:**
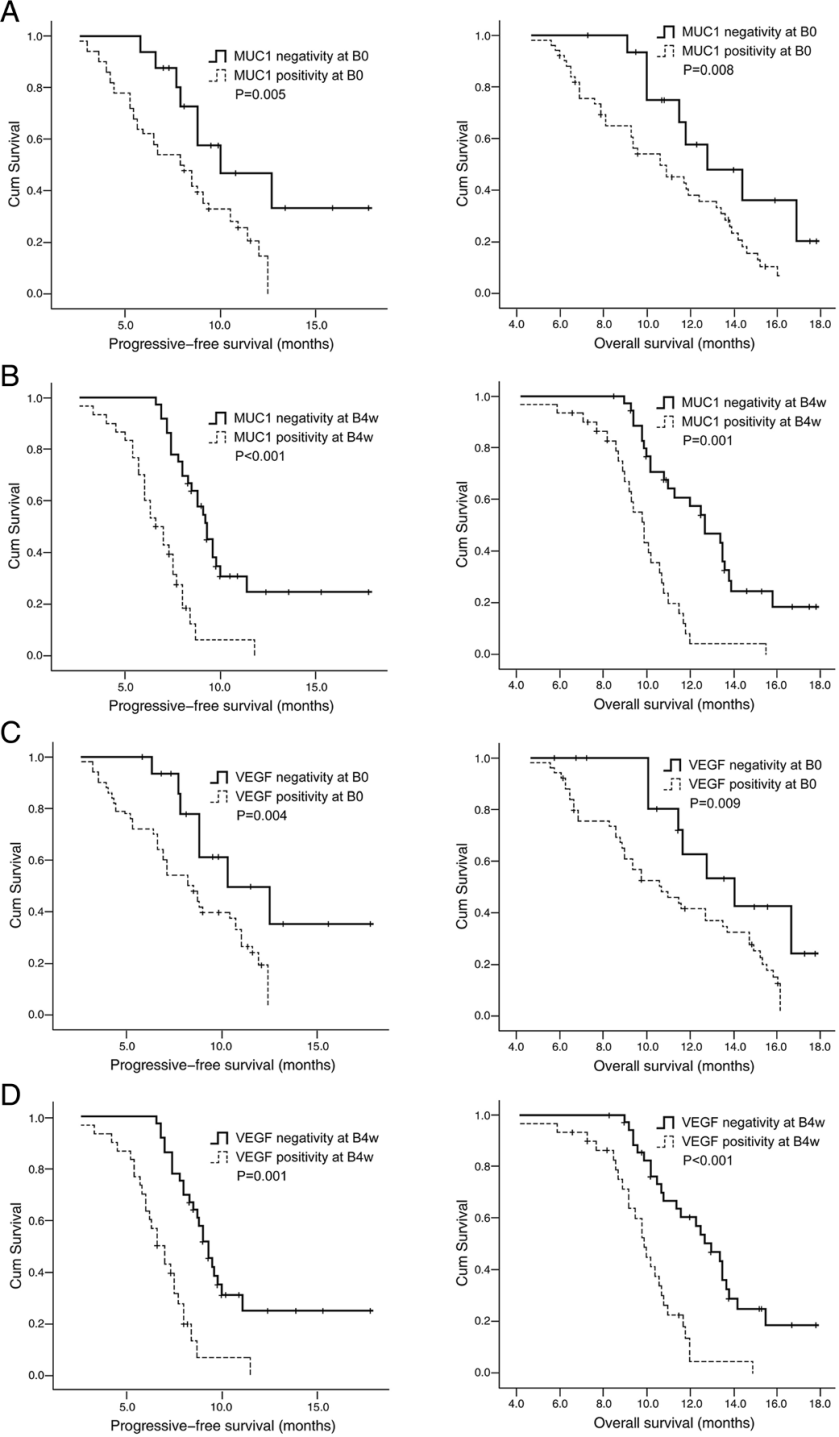
**Kaplan-Meier curves of progression-free survival (PFS) and overall survival (OS). (A and B)** PFS and OS curves according to the positivity or the negativity of MUC1 mRNA in blood of NSCLC patients before (B0) and 4 weeks after the start of treatment (B4w). **(C and D)** PFS and OS curves according to the positivity or the negativity of VEGF mRNA in blood of NSCLC patients at B0 and B4w time points.

Survival was analyzed in the all 66 patients, the median follow-up time was 11.2 months [95% confidence interval (CI): 8.4-16.6]. At the time of analysis, 48 patients had died and 18 patients had survived. For the entire patient population, the median PFS and OS were 5.2 months (95% CI: 2.6-8.9) and 10.8 months (95% CI: 7.3-15.2) respectively. Patients with blood MUC1 mRNA positivity at B0 and B4w proved to have significantly shorter median PFS and OS when compared with patients presenting with blood MUC1 mRNA negativity (P = 0.005 and P = 0.008, respectively at B0; and P < 0.001 and P = 0.001, respectively at B4w; Figure 3A and B). The similar results were found in patients with VEGF mRNA positivity and negativity at two sampling time points (P = 0.004 and P = 0.009, respectively at D0; P = 0.001 and P < 0.001 respectively at D4w; Figure 3C and D).

Univariate analysis showed that adenocarcinoma histology, EGFR mutations, and blood MUC1 and VEGF mRNA positivity were associated with PFS. PS, disease stage, adenocareinoma histology, EGFR mutations and the two marker positivity were associated with OS. A multivariate Cox proportional hazard model for PFS and OS was built using the variables that were found significant at the univariate analysis. Blood MUC1 and VEGF mRNA positivity at two sampling time points and EGFR mutation were independent predictors of shorter PFS (Table 4). Furthermore, blood MUC1 and VEGF mRNA positivity, PS and EGFR mutation were independent predictors of wore OS (Table 4).

**Table 4:** **Multivariate Cox proportional hazard model analysis of PFS and OS**

Variable	Progression-free survival			Overall survival		
	Hazard ratio	95% CI	P value	Hazard ratio	95% CI	P value
Histology						
ADC vs non-ADC	1.154	0.637-2.622	0.255	1.118	0.563-2.527	0.248
Performance status						
0-1 vs 2-3	1.845	1.052-2.995	0.114	2.532	1.219-4.325	0.013
Disease stage						
IIIB vs IV	0.875	0.317-2.152	0.416	1.272	0.428-2.257	0.167
EGFR status						
Wild-type vs mutation	2.726	1.415-4.655	0.011	2.615	1.338-4.524	0.014
MUC1 mRNA at B0						
Positivity vs negativity	2.359	1.155-4.326	0.018	2.494	1.536-4.721	0.015
MUC1 mRNA at B4w						
Positivity vs negativity	2.855	1.512-4.779	0.007	2.842	1.975-5.013	0.009
VEGF mRNA at B0						
Positivity vs negativity	2.453	1.415-4.592	0.013	2.577	1.482-4.683	0.011
VEGF mRNA at B4w						
Positivity vs negativity	3.012	2.103-5.148	0.004	2.910	1.971-5.106	0.006

ADC, adenocarcinoma.

## Discussion

Several markers have been identified that predict response to the EGFR-TKIs in NSCLC patients. Among them, EGFR mutation status was found to be the strongest predictive marker for the response to EGFR-TKIs and survival [7–9, 12–14]. Meanwhile, emerging data suggest that resistance to EGFR-TKIs may be also due to the activation of protein downstream of the receptor (K-RAS, mitogen-activated protein kinase, and signal transducers and activators of transcription 3), epithelial-mesenchymal transition of tumor cells, and other cell surface proteins, such as cMET [22–26]. Nevertheless, all these changes do not completely explain the variable clinical outcomes, and identification of other biomarkers of EGFR-TKI sensitivity/resistance may help in optimal patient selection.

Previous studies have reported significant associations between serum MUC1 and VEGF levels and tumor response, PFS and OS in patients with advanced NSCLC treated with EGFR-TKIs [16, 17]. By using the highly sensitive RTQ-PCR assay in a representative series of NSCLC patients, we demonstrate that detections of MUC1 and VEGF mRNA in peripheral blood are valuable diagnostic tools to identify a subset of NSCLC patients who benefit from gefitinib treatment.

MUC1 is a cell surface glycoprotein and aberrantly overexpressed in various carcinomas of epithelial origin including NSCLC, and induce gene signatures that are associated with poor survival of NSCLC patients [27]. MUC1 is translated as a single polypeptide that undergoes autocleavege into MUC1-N and MUC1-C subunits. MUC1-C is a transmembrane protein that functions as a cell surface receptor [28]. The MUC1-C extracellular domain interacts with ligand galectin-3 and thereby forms complexes with EGFR [28]. The available evidence indicates that MUC1-C is a binding partner and a substrate of EGFR, and it expression can promote EGFR-mediated signaling, while also enhancing EGFR stability by inhibiting its down-regulation upon EGFR stimulation [29, 30]. In addition, MUC1-C activates the phosphatidylinositol3-kinase (PI3K)-Akt pathway and the MUC1-C cytoplasmic domain has an YHPM site that following phosphorylation functions as a binding site for the PI3K SHZ domain [31]. Some studies have indicated that effective treatment of NSCLC cells with EGFR inhibitors is associated with suppression of PI3K activity and resistance to these inhibitors occurs with reactivation of the PI3K-Akt signaling pathway [32]. Overexpression of MUC1 as found in human carcinomas is associated with accumulation of MUC1-C in cytoplasm and targeting of MUC1-C to the nucleus [27]. Although the exact role of blood MUC1 in development and progression of NSCLC has not been completely illuminated, these findings suggest that MUC1 can influence EGFR signaling directly by binding with EGFR or indirectly through it interaction with PI3K-Art pathway, regulating the clinical efficacy of EGFR-TKI treatment.

In the present study, we show that blood levels of MUC1 mRNA were dramatically decreased during EGFR-TKI treatment. But blood MUC1 mRNA remained positivity in 45.5% of these NSCLC patients at 4 weeks after EGFR-TKI treatment. Moreover, the blood levels of MUC1 mRNA during treatment were significantly increased in patients with tumor response of PD, whereas the patients who achieved a PR had a significant decrease in blood MUC1 mRNA levels, implying that the changes of MUC1 mRNA levels in the course of treatment with gefitinib may predict imaging response to treatment. Furthermore, the positivity of blood MUC1 mRNA before and during EGFR-TKI treatment were significantly associated with shorter PFS and OS, which were further demonstrated by multivariate analysis. Our results were in line with the study by Ishikawa et al. and showed that blood MUC1 detection could be used as a marker to predict the efficacy of gefitinib treatment in patients with advanced NSCLC [16].

VEGF is a critical proangiogenic factor in tumor and promotes endothelial cell growth, survival, and migration and mediates vessel permeability, thereby facilitating tumor progression and metastatic spread [33]. The VEGF and EGFR pathway are closely related, sharing common down-stream signaling pathway [34]. EGF and transforming growth factor-α both induce VEGF expression via activation of EGFR in cell culture models and have proangiogenic properties. EGFR pathway modulates angiogenesis by up-regulating VEGF or other key mediators in angiogenic process [34]. In preclinical models, EGFR blockade with the monoclonal antibody cetuximab resulted in down-regulation of proangiogenic mediators, including VEGF, accompanied by reductions in microvessel density and metastasis [35]. On the basis of these data, we hypothesize that blood VEGF mRNA levels have the potential to be a predictive marker of clinical benefit in patients with advanced NSCLC treatment with EGFR-TKIs.

In the present study, we showed that the positivity of VEGF mRNA in blood samples detected by the RTQ-PCR assay was correlated statistically with responsiveness to, and the PFS and OS of, gefitinib treatment. Moreover, our study have also shown a relationship between the changes of VEGF mRNA levels on the course of gefitinib treatment and imaging response, which is similar to association between the changes of MUC1 mRNA levels and imaging response to gefitinib.

In the study by Kasahar et al. [17], the pretreatment serum VEGF levels were measured in 95 patients with lung adenocarcinoma who received EGFR-TKI treatment, although patients presenting with a higher serum VEGF levels proved to have a poor tumor response, significantly shorter PFS and OS than patients with lower serum VEGF levels, these features did not independently determine OS in multivariate analysis. A possible reason of the discrepancy between the study by Kasahar et al. and our study may be due to different method of VEGF detection. Kasahar et al. used enzymelinked immunosorbent assay (ELISA) to measure serum VEGF levels, while we applied RTQ-PCR technique to detect blood VEGF mRNA expression. We infer that VEGF mRNA detected by RTQ-PCR was more sensitive and accurate than serum VEGF level measured by ELISA.

We are aware of some limitations in the present study. First, the total sample size is relatively small which may result in some bias of result. Second, blood MUC1 and VEGF mRNA levels detected at two sampling time points did not be compared with CEA and CYFRA 21–1 which generally recognized as two prognostic markers for NSCLC. Third, detection of MUC1 and VEGF mRNA using RTQ-PCR is relatively complicated in methodology and experimental handle is time--consuming which may influence routine use in clinical practice, although RTQ-PCR is a highly sensitivity and specific analysis tool.

## Conclusions

In summary, our results show that NSCLC patients with positivity of blood MUC1 and VEGF mRNA seem to have poor outcomes with gefitinib treatment, in terms of PFS, OS and response, than those with negativity of the two markers. These findings support the nation that the detection of blood MUC1 and VEGF mRNA by RTQ-PCR could to be used as biomarkers to predict treatment efficacy of EGFR-TKIs in NSCLC patients. Further study with large number of patient is warranted to clarify the clinical utility of RTQ-PCR assay for MUC1 and VEGF mRNA expression in blood sample in determination of the optimal treatment for advanced NSCLC patients.

## Abbreviations

BLD: Benign lung disease
CR: Complete response
EGFR: Epidermal growth factor receptor
CT: Computerized tomography
MUC1: Mucin1
NSCLC: Non-small cell lung cancer
OS: Overall survival
PBMCs: Peripheral blood mononuclear cells
PD: Progressive disease
PFS: Progression-free survival
PR: Partial response
PS: Performance status
PI3K: Phosphatidylinositol3-kinase
RTQ- PCR: Real-time quantitative-Polymerase chain reaction
SD: Stable disease
TKIs: Tyrosine kinas inhibitors
VEGF: Vascular endothelial growth factor.
